# Effect of Cationic Modified Microcrystalline Cellulose on the Emulsifying Properties and Water/Oil Interface Behavior of Soybean Protein Isolate

**DOI:** 10.3390/foods11193100

**Published:** 2022-10-05

**Authors:** Yunsi Guo, Sirui Feng, Zhangpeng Li, Minghao Jiang, Zile Xiao, Lichun Chen, Yue Zhang

**Affiliations:** 1 School of Food Science and Biotechnology, Zhejiang Gongshang University, Hangzhou 310018, China; 2 Food Nutrition Science Centre, Zhejiang Gongshang University, Hangzhou 310012, China

**Keywords:** cationic modification, microcrystalline cellulose, charge density, SPI–polysaccharide complexes, emulsifying properties, rheological properties

## Abstract

Stabilizing emulsion using complex biopolymers is a common strategy. It would be very interesting to characterize the impact of charge density on the emulsifying properties of complex polyelectrolytes carrying opposite charges. In this study, cationic modified microcrystalline celluloses (CMCC) of different charge densities were prepared and mixed with soy protein isolate (SPI) for emulsion applications. CMCC-1 to 3 with various cationic charge values were successfully prepared as characterized by zeta-potential and FTIR. The positive charge density’s effects on solubility, thermogravimetric properties, and rheological properties were studied. Complexes of SPI-CMCC with various zeta-potential values were then obtained and used to stabilize soybean oil emulsions. The results show that emulsions stabilized by complexes of SPI and CMCC-3 at a ratio of 1:3 had the best emulsification ability and stability. However, the interfacial tension-reducing ability of complexes decreased continuously with increasing cationic charge value, while the rheological results show that complexes of SPI-CMCC-3 at a ratio of 1:3 formed a stronger viscoelastic network than other complexes. Our results indicate that this SPI-CMCC complex formula showed excellent emulsification performance, which could be adjusted and promoted by changing the charge density. This complex formula is promising for fabrication of emulsion-based food and cosmetic products.

## 1. Introduction

Soybean protein isolate (SPI) is a by-product of soybean oil production and a common food ingredient, filling roles such as emulsifier and gelling agent. It is believed that SPI could reduce oil–water interfacial tension, provide a physical barrier between the two phases, and therefore stabilize emulsions [1]. However, like other protein-based emulsifiers, SPI is very sensitive to environmental factors, such as pH, temperature, and ionic strength, which seriously affect the stability of SPI-based emulsions [2]. For example, when the pH value is close to the isoelectric point of the protein, the emulsion stabilized by SPI is prone to aggregation and sedimentation, thus limiting its application as an emulsifier [3].

Complexation or conjugation with polysaccharides could greatly improve the emulsifying properties of SPI [4]. For example, the complexes of SPI with soy hull polysaccharides could form a viscoelastic film at o/w interface and significantly improve the stability of emulsions by slowing down the motion of droplets [5]. The Pickering emulsion fabricated by using SPI and chitosan complexes was stable against high ionic strength and temperature [6]. For these SPI–polysaccharide complexes, the electrostatic interaction between them has been considered as one of the main driving forces for the formation of stable complexes and responsible for the better emulsion stability. For instance, Zhang et al. [7] found that Pickering emulsion stabilized by SPI and oxidizing bacterial cellulose showed the best stability at pH 3, at which condition these two components could form electrostatic complexes. However, there are still many unknown issues with the formation of complexes between proteins and polysaccharides, the stabilization mechanisms of complexes on water–oil interface, and also the determining factors. Some studies have studied the effects of polysaccharides with different charges on the emulsifying performance of SPI [8,9]. Unfortunately, the selected polysaccharides showed completely different structures, origins, and even purities, which may not be able to reflect the impacts of charge density on the complexation and stabilization of o/w interface directly. It would be more convincing to modify a polysaccharide with various charge density, obtain the complexes with different charge values, and then compare their interfacial behaviors and emulsifying properties to illustrate the stabilizing mechanism. SPI carries negative surface charge at neutral pH (such as pH 7), which requires a polyelectrolyte with positive charge to form a complex with SPI [10]. However, chitosan is the only natural polysaccharide that carries positive charges, and thus, cationic modification on polysaccharide is necessary for the complexation with SPI at this pH condition.

Microcrystalline cellulose (MCC), one of the most common forms of cellulose derivatives, is mainly obtained by the partial hydrolysis of amorphous regions in natural cellulose [11]. Due to its good biodegradability and biocompatibility, MCC has been used as encapsulation material for a variety of drug molecules for oral delivery purpose [12,13]. There are abundant hydroxyl groups contained in MCC, which can be easily modified according to demands [14]. For example, a hydroxyl group could be oxidized to carboxylic acid by TEMPO (2,2,6,6-tetramethylpiperidine-1-oxyl radical) [15] or grafted to glycan trimethylammonium chloride to introduce positive charge [16], or a series of cellulose esters and ethers could be obtained [17]. Compared with other heteropolysaccharides, MCC shows a linear and clear structure, so it is suitable to be used as a model polysaccharide to be modified into cationic polysaccharides and to study the impacts of charge density on the complexation ability with SPI carrying negative charge and therefore their interfacial behavior and emulsification performance.

The main objectives of this study were to prepare and characterize cationic modified MCC (CMCC) with different charge densities; fabricate complexes with SPI to stabilize oil-in-water emulsion; analyze the droplet size, zeta-potential, and emulsifying ability; compare and explore the influence of CMCC with different charges on the complexation with SPI and the resultant interfacial behavior and emulsifying performance. These results could provide a clearer understanding on the role of surface charge density in the formation of protein–polysaccharide complexes and the stabilizatiodn mechanism of these complexes on water–oil interface, which is useful for the screening of protein–polysaccharide complexes for food emulsion applications.

## 2. Materials and Methods

### 2.1. Materials

Soybean protein isolate (SPI), microcrystalline cellulose (MCC), 2,3-epoxypropyl trimethyl ammonium chloride (GTMAC), dimethyl sulfoxide (DMSO), and soybean oil were all purchased from Shanghai Maclean Biochemical Technology Co., Ltd. (Shanghai, China), and ethanol was bought from Xilong Scientific Co., Ltd. (Guangzhou, China). All chemicals and reagents used in this study were analytic grade.

### 2.2. Cationic Modification of Microcrystalline Cellulose

**Scheme 1:**
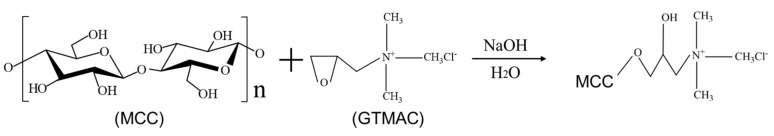
Synthetic route for the preparation of CMCC.

As shown in Scheme 1, MCC was modified by cationic modification according to the method of Zaman et al. [15]. MCC and NaOH (5% by mass of MCC) were dispersed in a water/DMSO mixture (36% *v*/*v* water), and then GTMAC was added, which was three times the mass of the dehydrated glucose unit. The dispersion was firstly sonicated for 30 min and then stirred in a water bath at 65 °C for 2 h. The reaction was terminated within 95% ethanol and then centrifuged and washed for three times using 95% ethanol. Sample CMCC-3 was obtained.

In another set, MCC and NaOH (0.5% of MCC mass) were dispersed in the mixture of water/DMSO with addition of GTMAC, as stated above. The modification reaction was conducted in a water bath at 65 °C for 10 min and 20 min, respectively, and then terminated. After centrifugation and washing, two samples, named CMCC-1 and CMCC-2, were obtained. The obtained samples were re-dissolved in deionized water and dialyzed for 48 h.

### 2.3. Characterization of CMCC

#### 2.3.1. Measurement of Surface Charge and Solubility

MCC and CMCC were dispersed in deionized water at 0.1% *w*/*v*, and zeta-potential values of all samples were then determined using a dynamic light-scattering instrument (Litesizer 500, Anton Paar, Graz, Austria). The samples are named as MCC, CMCC-1, CMCC-2, and CMCC-3 with the increasing magnitude of zeta-potential.

The solubility of samples in water was determined according to the method of Hao et al. [17]. A sample of about 20 mg was accurately weighed and dissolved in 4 mL deionized water in a centrifuge tube. After vortexing using a whirlpool mixer (Vortex 2, IKA, Staufen, Germany), all samples were centrifuged at 12,000× *g* for 10 min. The supernatant was removed and lyophilized with a final dry weight of $W_{s}$. The solubility can be calculated as follows:

(1) $$solubility\left(\%\right)=\frac{W_{s}}{W}\times 100$$

where $W_{s}$ and W are the mass values of sample after lyophilization and total mass values of sample, respectively.

#### 2.3.2. Fourier Transform Infrared Spectroscopy (FTIR) Analysis

The absorption spectra of the lyophilized samples were recorded using Fourier infrared spectrometer (iS50, Nicolet, Waltham, MA, USA) at 4000–400 cm^−1^ with a resolution of 4 cm^−1^ and scanned 64 times. The representative curve is the average of three independent replicates.

#### 2.3.3. Thermogravimetric (TG) Analysis

According to the method of Huang et al. [18], thermogravimetric analyzer (TGA2, Mettler Toledo, Greifensee, Switzerland) was used to conduct thermogravimetric analysis of the samples. The measurement was performed under a nitrogen atmosphere at a flow rate of 20 mL/min. The test temperature range was set as 30~500 °C, and the heating rate was 10 °C/min.

#### 2.3.4. Rheological Properties Analysis

Rheological measurements were performed using a MCR 302 rotary rheometer (Anton Paar, Graz, Austria). All experiments were performed at 25 °C using a parallel-plate (PP50) geometry with a measuring gap of 0.5 mm. The concentration of MCC/CMCC was 2% (*w*/*v*), and strain tests were performed in the range of 0.1~50% at a frequency of 1 Hz. Viscosity tests were performed at a shear rate of 0.1~100 s^−1^.

### 2.4. Preparation of Composite Emulsions Stabilized by Complexes of SPI and CMCC

SPI was dispersed with MCC, CMCC-1, CMCC-2, and CMCC-3 in deionized water and adjusted to pH 7.0 using 1 M HCl or NaOH to increase the solubility of SPI and also avoid any negative effect at acidic pH. The mixtures were stirred overnight for full hydration of both protein and CMCC samples. SPI and MCC/CMCC dispersions were mixed on the basis of a certain proportion of weight to obtain SPI-MCC/CMCC complex dispersions of different proportions. Then, emulsions were made by homogenizing 20% (*v*/*v*) soybean oil into above dispersions at 15,000 rpm for 5 min using a high-speed shear homogenizer (T18, IKA, Staufen, Germany). The mass proportions of protein and MCC/CMCC in the final emulsion were 3:1, 1:1, and 1:3, respectively, with a total concentration of 1% (*w*/*v*).

### 2.5. Characterization of Emulsions

#### 2.5.1. Measurement of Droplet Size, Zeta-Potential, and Morphology

The droplet size of emulsions was determined by laser particle size analyzer (Microtrac Sync, Haan, Germany). The refractive index of the emulsion was set to 1.47, while water (refractive index of 1.33) was used as dispersant. The size of the emulsion droplet was expressed as the volume-weighted mean diameter (d_4,3_). The zeta-potential of emulsions was measured using above dynamic light-scattering instrument.

Emulsion (20 µL) was dropped onto the slide to ensure that there were no bubbles. After pressing on the cover slide, the morphology of the droplets was observed by magnification of 100X with a light microscope (DM4, Leica, Wetzlar, Germany).

#### 2.5.2. Measurement of Emulsification Index

The emulsification index (EI) was determined according to the method of Zhang et al. with some modifications [19]. The freshly prepared emulsion (4 mL) was placed in a centrifuge tube and sealed at room temperature. The total height of the emulsion and the height of the upper layer of creaming were measured within 7 days. The formula of EI is shown below:

(2) $$EI(\%)=\left(\frac{H_{c}}{H_{t}}\right)\times 100$$

where $H_{c}$ and $H_{t}$ are the height of creaming layer and the total height of emulsion, respectively.

### 2.6. Interfacial Behavior and Interaction Mechanism

#### 2.6.1. Measurement of Interfacial Tension

The interfacial tension of O/W interface was measured by sessile drop method using goniometer (DSA 25, KRÜSS, Hamburg, Germany) [20]. SPI was mixed with MCC, CMCC-1, CMCC-2, and CMCC-3 in deionized water at a series of MCC/CMCC ratios of 3:1, 1:1, and 1:3 (*v*/*v*) and stirred overnight to fully hydrate at a final concentration of 0.2% (*w*/*v*). The dispersion was loaded into a micro-syringe, and a drop of 20 µL sample was dripped into a square glass cylinder with soybean oil through a stainless steel needle (with an outer diameter of 1.8 mm) to form hanging drops. The interfacial tension was monitored for 5 min to evaluate the change in the oil–water interface. The interfacial tension of SPI dispersion was measured by the same method as a control.

#### 2.6.2. Measurement of Rheological Properties

The complex dispersions of SPI and MCC/CMCC at a ratio of 1:3 were prepared at a total mass concentration of 2% (*w*/*v*). The viscosity of dispersions with a shear rate range of 0.1~100 s^−1^ was measured, while the storage and loss moduli within a strain range of 1~50% and a frequency of 1 Hz were studied.

#### 2.6.3. Fluorescence Quenching

The changes in SPI structure after interaction with CMCC were explored by fluorescence quenching method using a fluorescence photometer (F-4700, Hitachi, Tokyo, Japan) [21]. SPI (1 mg/mL) and MCC, CMCC-1, CMCC-2, and CMCC-3 stock dispersions (40 mg/mL) were prepared at pH 7.0 and stirred overnight. The excitation wavelength was set at 280 nm, and the emission within the range of 300~500 nm was measured. The excitation slit was 5 nm, emission slit was 10 nm, and scanning speed was 1200 nm/min. Two ml of SPI dispersion was loaded into a quartz cuvette, and the dispersion of MCC/CMCC was titrated into it with a concentration gradient of 0.2 mg/mL until a final concentration of 2 mg/mL was achieved. The experiment was carried out at 25 °C. The fluorescence values of pure water and MCC/CMCC dispersions were subtracted as background.

Fluorescence quenching data were analyzed by Stern–Volmer equation as below:

(3) $$\frac{F_{0}}{F}=1+K_{SV}\left[Q\right]$$

where $F_{0}$ and F are the fluorescence intensity of SPI in the absence and presence of quencher (MCC/CMCC), *Q* is the concentration of MCC/CMCC, and *K*_SV_ is the Stern–Volmer quenching constant.

### 2.7. Statistical Analysis

All experiments were performed with at least three replicates. Results are expressed as mean ± standard deviation, unless otherwise stated. SPSS 26.0 was used for statistical analysis, and *t*-test was used for one-way ANOVA with significance level (*p*) of 0.05.

## 3. Results and Discussion

### 3.1. Characterization of CMCC

**Figure 1:**
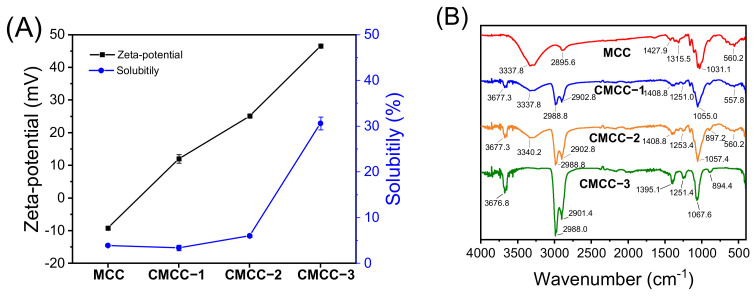
(**A**) The zeta-potential and water-solubility and (**B**) FTIR measurement of MCC and CMCC.

The cationic reagent GTMAC can be used for the modification of microcrystalline cellulose [15,18], that is, through this reagent, quaternary ammonium groups can be grafted onto MCC, providing positive charge on the surface. The surface charge of modified MCC was monitored by zeta-potential measurement, while the water solubility of all modified samples was also studied. As shown in Figure 1A, MCC showed very low electronegativity with a value of −9.2 mV. After introducing the quaternary ammonium group, the surface charge rose to 12.0~46.5 mV. The zeta-potential measurement also indicated that the grafting degree of quaternary ammonium group gradually increased with reaction time. A higher surface charge could also be achieved by high content of NaOH (such as CMCC-3 sample) [22]. The solubility of MCC was only 3.9%, due to strong intramolecular hydrogen bonds between MCC polymeric chains [23]. With the increase in surface charge, the solubility of CMCC dispersions also increased. When the exposed hydroxyl group on the surface of MCC was modified into quaternary ammonium groups, the resultant strong intermolecular electrostatic repulsions between polymeric chains separated the polymers from each other and therefore enhanced the water solubility [24,25].

The Fourier transform infrared (FTIR) spectra of MCC and CMCC are shown in Figure 1B. The absorption peak around 3337 cm^−1^ usually represents the stretching of hydroxyl group and hydrogen bond interaction [26]. For MCC, the intensity of this peak was strongest. With the increase in grafting degree, the hydroxyl group in CMCC was constantly replaced, and the peak intensity was gradually decreased. The absorption peak at 2988 cm^−1^ represents the C-H stretching vibration from -CH_2_ and -CH_3_ [18]. The peak intensity increased obviously with the grafting of the quaternary ammonium group in CMCC. The fluctuation at 1640 cm^−1^ was attributed to the adsorption of water molecules [27]. In addition, the peak around 1400 cm^−1^ was attributed to the change in *δ*_C-H_ and the methyl group of quaternary ammoniums introduced during cationic modification. With the increase in grafting degree, the intensity of this peak was significantly enhanced. All the above observations indicated the successful synthesis of CMCC [15,28]. The FT-IR results are consistent with the zeta-potential measurements.

**Figure 2:**
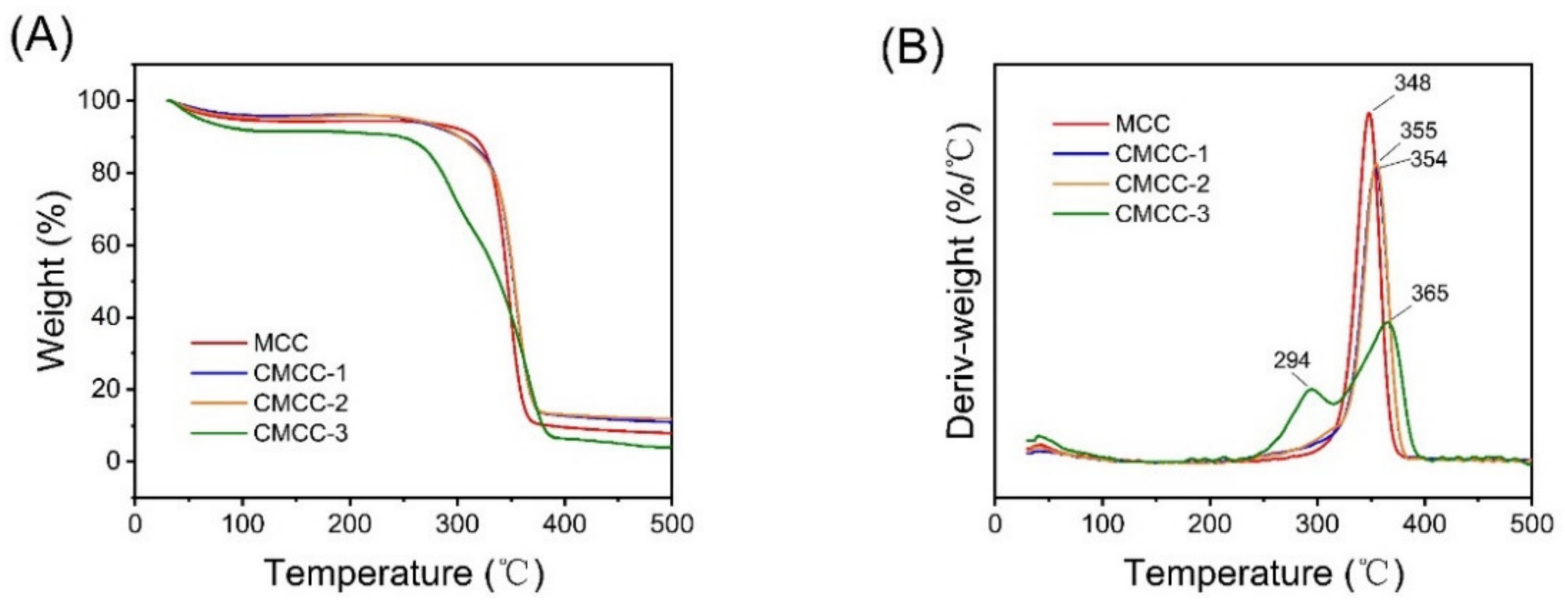
The TGA (**A**) and DTG (**B**) curves of MCC and CMCC.

Thermogravimetric analysis (TGA) was also conducted to study the thermal stability of MCC and CMCC (Figure 2). The TGA results show that the thermal stability of the samples first increased and then decreased with the increasing degree of surface cationization (Figure 2A). The initial weight loss of the differential thermogravimetry (DTG) curve in Figure 2B was due to evaporation of water. When heated to 348 °C, MCC, CMCC-1, and CMCC-2 had only one peak, while CMCC-3 showed a bimodal pattern. The shoulder peak at 294 °C of CMCC-3 may be associated with the thermal decomposition of quaternary ammonium groups and ether linkage between cellulose and derivatization groups [28]. After cationization, the decomposition temperature of cellulose itself increased gradually from 348 to 354 °C, 355 °C, and 365 °C, confirming the structure change in the MCC crystalline region after cationic modification by GTMAC. The results also show that MCC, CMCC-1, and CMCC-2 had similar TGA and DTG patterns, but CMCC-3 had a different curve. In other words, the crystalline region of CMCC-3 was significantly changed after modification.

**Figure 3:**
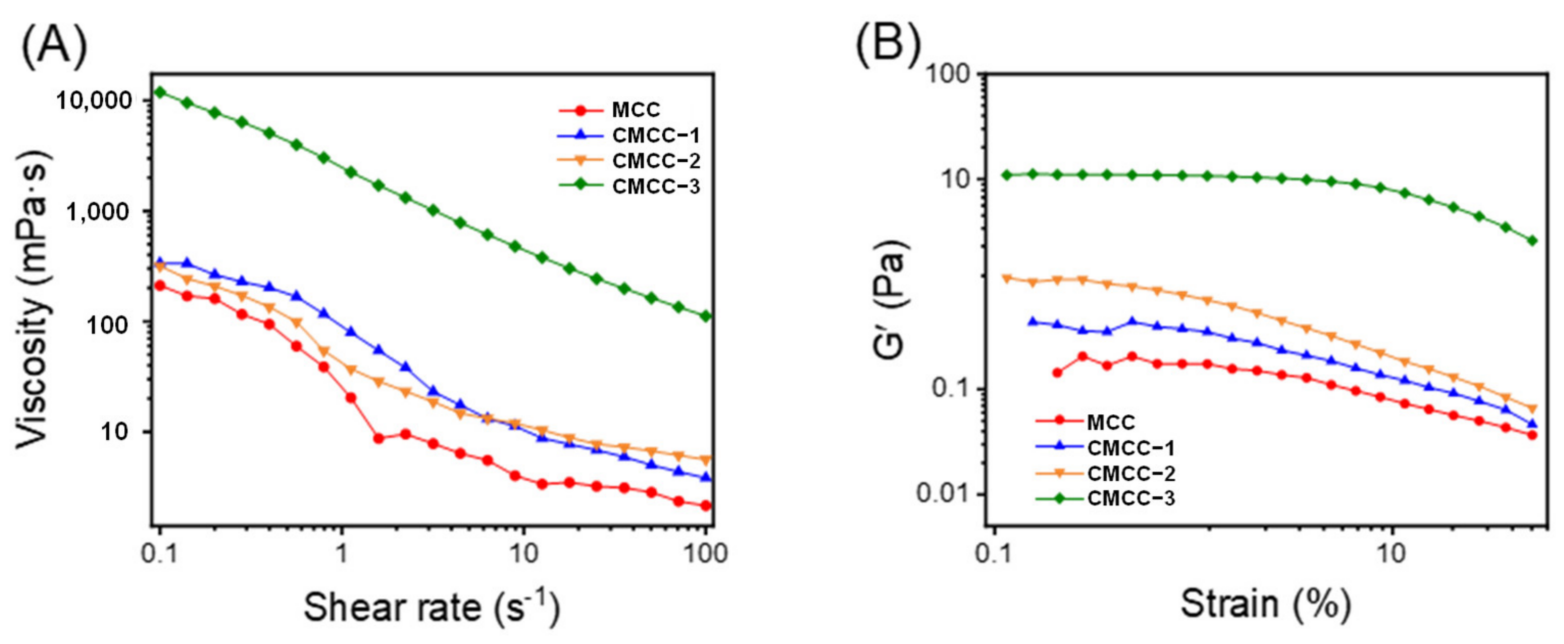
The (**A**) shear viscosity and (**B**) storage modulus (G’) of MCC and CMCC in a strain sweep test.

The rheological properties of CMCC were also studied. As shown in Figure 3A, the viscosity of all sample dispersions decreased continuously with shear rate, showing typical shear thinning behavior and pseudoplastic fluid characteristics [29]. When shear stress was applied, MCC tried to align in the direction of the shear flow [30]. The viscosity of all CMCCs was greater than that of MCC in the whole shear rate range and increased with the increase in cationization degree. This observation was consistent with solubility results that CMCC was more flexible in water than MCC. In particular, the viscosity of CMCC-3 was an order of magnitude greater than that of CMCC-1 and CMCC-2, with lower surface charge value. The results confirm our observations that the structure of sample CMCC-3 was completely different from the others. Figure 3B shows the storage modulus (G’) changes of the four samples as a function of strain. It can be seen that with the increase in cationization degree, the G’ of sample dispersions increased continuously, showing the increase in elastic characteristics. CMCC-3 showed a relatively wide linear viscoelastic region up to 10% strain, indicating that this sample showed much higher fracture modulus and mechanical strength. These results may also suggest that a higher degree of cationic modification could further increase the dispersibility of CMCC in water and form a more viscoelastic gel network in aqueous phase [26]. The G’’ changes of samples are shown in Figure S1. Sample CMCC-3 also showed a significantly higher viscous value than the other three samples, indicating a relatively more obvious viscous behavior. These results confirm that CMCC-3 showed significantly higher flexibility than other samples.

### 3.2. Emulsifying Properties of SPI-CMCC Complexes

The effect of CMCC with various charge densities on the emulsifying performance of SPI was studied by measuring the droplet size and zeta-potential of soybean oil emulsions. The results are shown in Table 1. The droplet size of all emulsions increased with the increase in MCC/CMCC proportion, except for SPI/CMCC-3 complexes. This may be caused by the co-adsorption of SPI and MCC/CMCC on the interface. Despite this CMCC-3 complex sample, the droplet size of emulsions decreased with increased degree of cationization, indicating that cationization was beneficial for better interaction and settlement of SPI with CMCC at interface. The incorporation of MCC and CMCC-1 could significantly increase the magnitude of zeta-potential of emulsions. For example, in the emulsion stabilized by SPI/MCC, the absolute value of zeta-potential increased from 41.1 mV to 45.3 mV with the increase in MCC content. However, for samples CMCC-2 and CMCC-3, with higher degree of cationization, the zeta-potential value of emulsions was moved to the opposite direction. Therefore, the emulsion stabilized by SPI/CMCC-3 at the proportion of 3:1 had a zeta-potential value close to zero (−2.4 mV), which could be the reason for the maximum droplet size of emulsion stabilized by this formula. The lack of electrostatic repulsion induced the coalescence of emulsion droplets. With the continuous increase in CMCC-3 in the formula, the charge density of the complexes increased gradually, and the droplet size of emulsion decreased significantly. The smallest droplet size was achieved by SPI/CMCC-3 at 1:3, which also had the largest magnitude of zeta-potential value. The droplet size of SPI/CMCC-3 at 1:1 was significantly smaller than that of SPI/MCC emulsion at the same proportion, while the absolute values of their zeta-potentials were the same. This observation may indicate that SPI/CMCC-3 complexes had better emulsifying properties, and electrostatic repulsion was not the only mechanism for the better stabilization performance.

**Figure 4:**
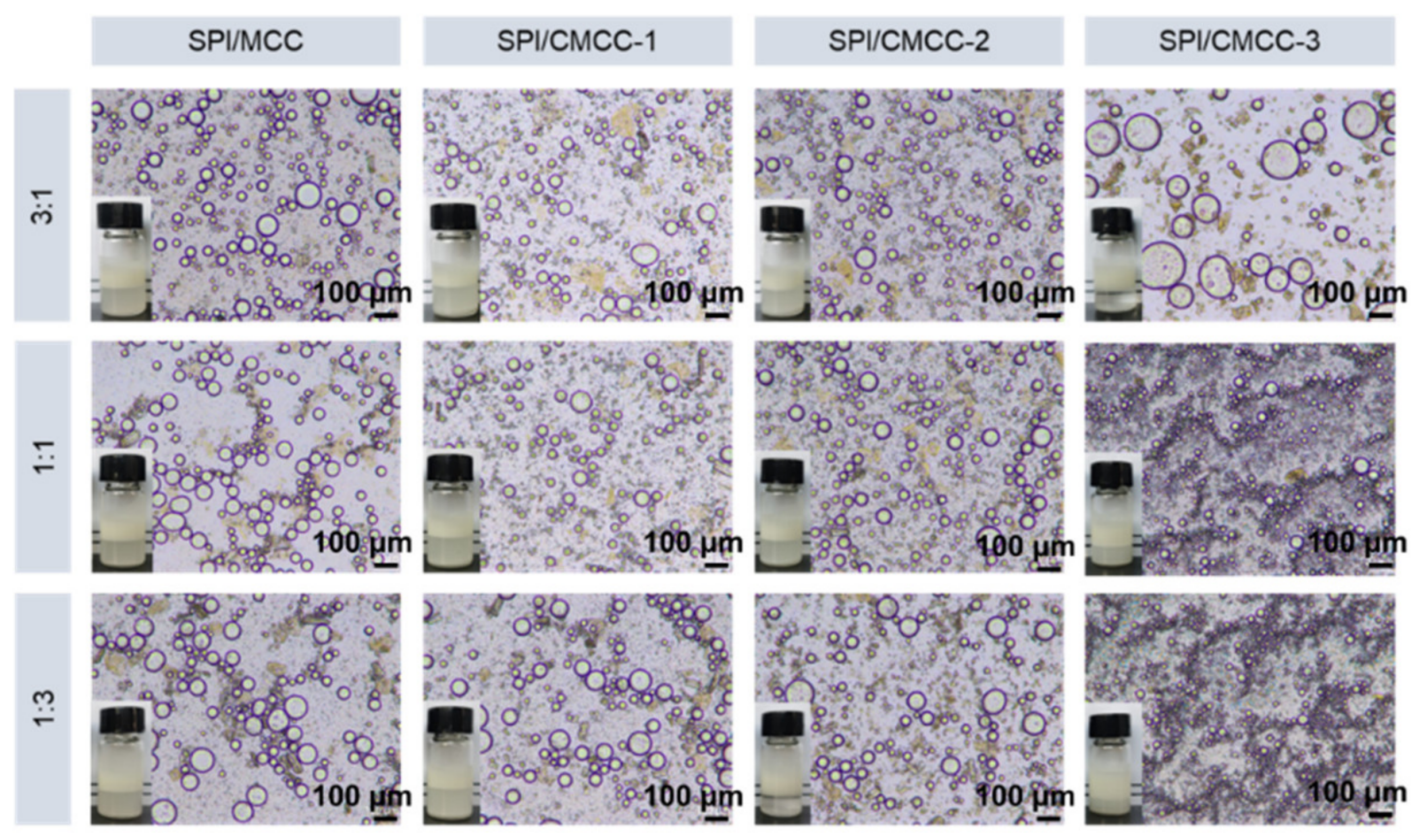
The microscope observation of emulsion stabilized by complexes of SPI and MCC/CMCC at ratios of 3:1, 1:1, and 1:3 (from up to down).

The microscopic observation of the emulsions is shown in Figure 4, which was consistent with the dynamic light-scattering measurement. Except for CMCC-3, the droplet size gradually increased with the increase in MCC/CMCC proportion. The existence of irregular and big oil droplets was obviously observed in sample SPI/CMCC-3 at the proportion of 3:1. The clustering of droplets can be observed in all emulsions, which can be explained by the simultaneous adsorption of one MCC or CMCC molecule on multiple droplet interfaces. Compared with other emulsions, the SPI/CMCC-3 emulsions contained a larger number of clustered droplets, which may be due to the stronger electrostatic interactions between CMCC-3 and SPI with opposite charges.

**Figure 5:**
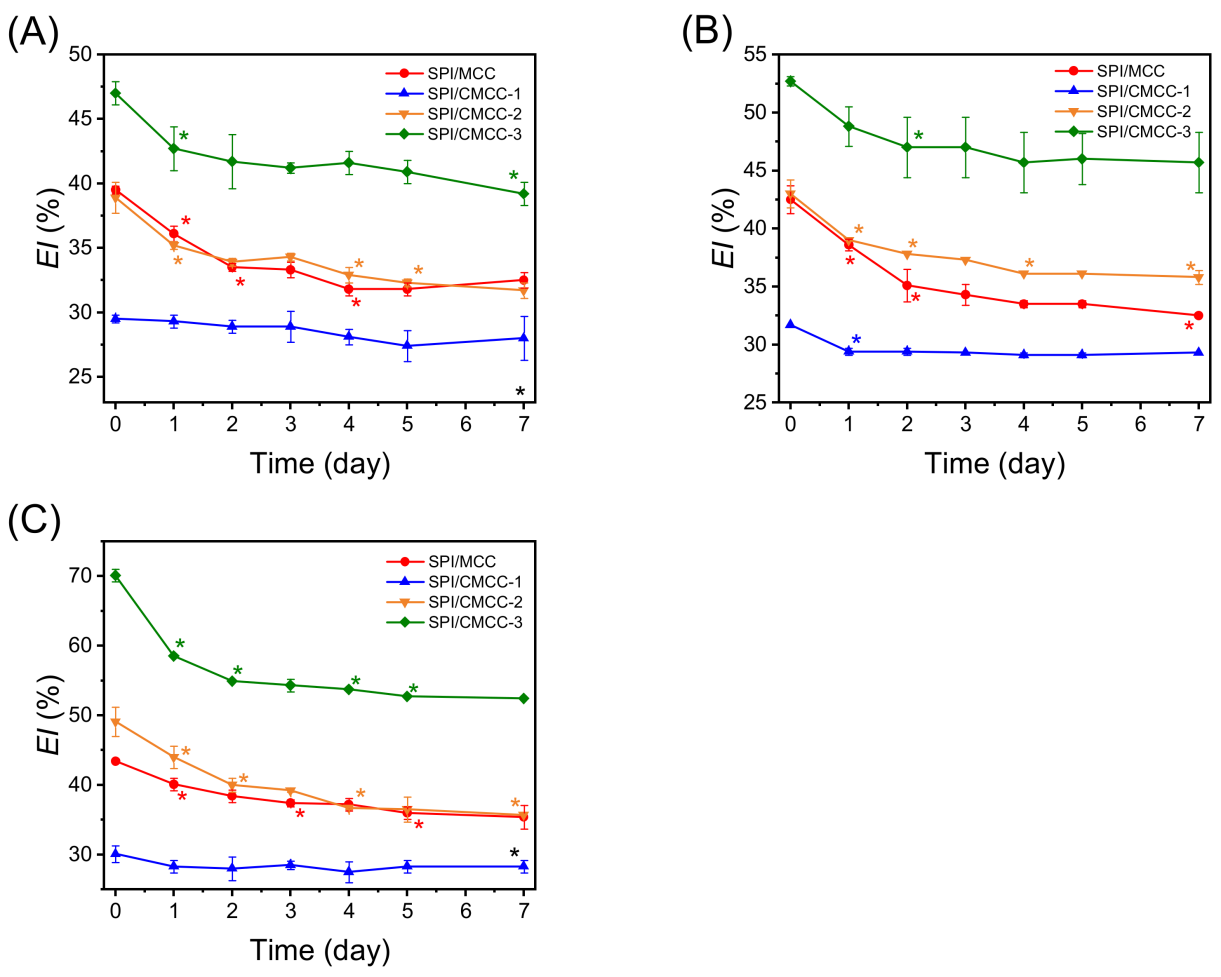
The emulsification index (EI) of the emulsion stabilized by complexes of SPI and MCC/CMCC at ratios of 3:1 (**A**), 1:1 (**B**), and 1:3 (**C**), * indicates significant difference from the previous data point (*p* < 0.05).

The emulsion stability was evaluated by measuring the change in EI during 7 days storage, and the results are shown in Figure 5. For all ratios, SPI/CMCC-1 stabilized emulsions had the lowest EI value. When comparing MCC with CMCC-2 and CMCC-3, the EI gradually increased with the increase in cationization degree, indicating that a higher degree of cationization was beneficial for the stability of complex emulsions. Although the droplet size of emulsion stabilized by SPI/CMCC-3 at a ratio of 3:1 was largest, this complex formula still showed a relatively large EI value, as evidence of high stability. This result indicates that the stability of the emulsion system in the presence of CMCC-3 could be mainly attributed to the formation of a gel-like network rather than electrostatic repulsive forces among droplets [31]. This phenomenon was also observed in other emulsions stabilized by fiber particles [19,32,33].

### 3.3. Interface Characteristics of SPI/CMCC Complexes and Their Interactions

**Figure 6:**
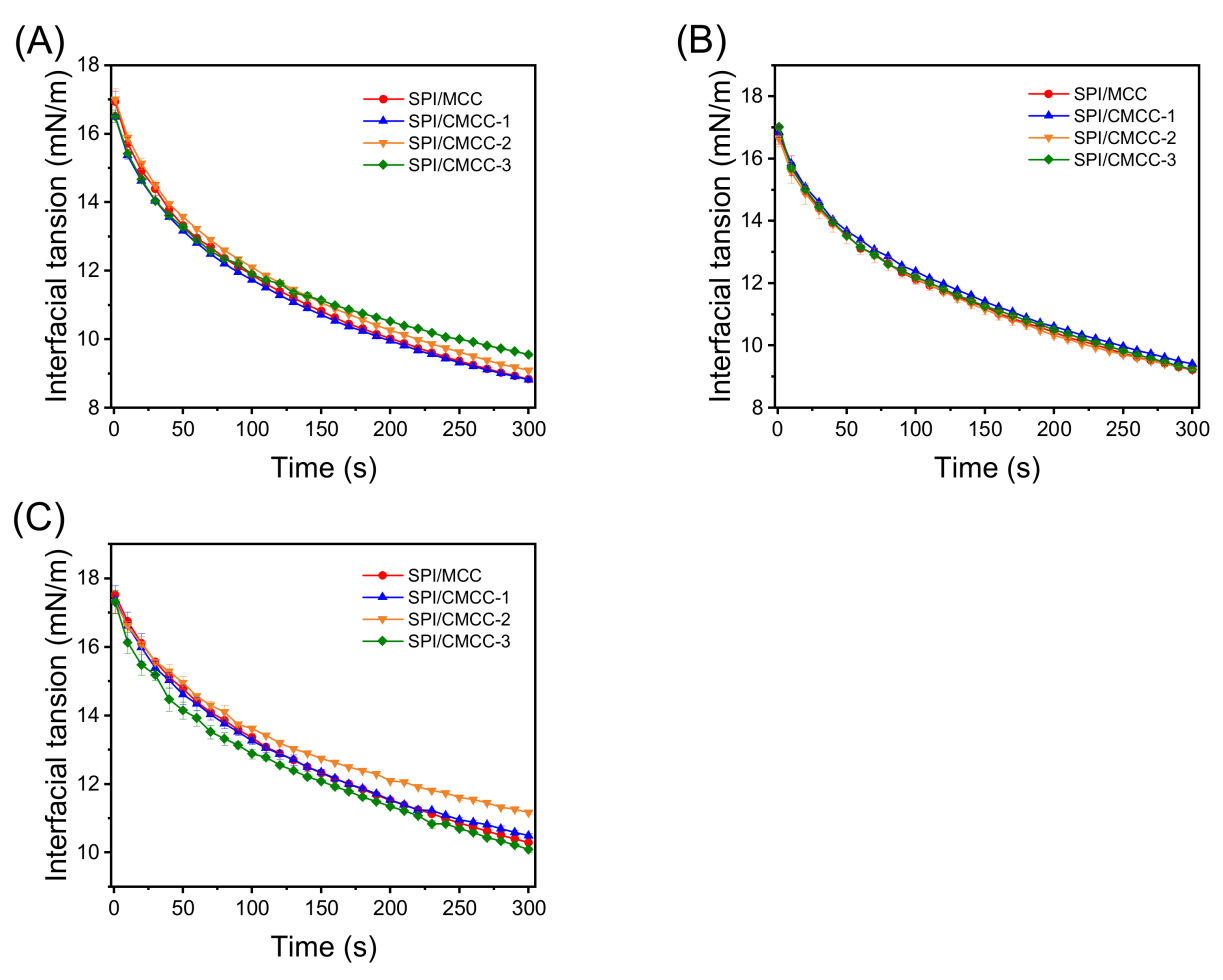
Interfacial tension of soybean oil/water interfaces as stabilized by complexes of SPI and MCC/CMCC at ratios of 3:1 (**A**), 1:1 (**B**), and 1:3 (**C**).

To better understand the behavior of the complexes of MCC/CMCC and SPI at O/W interface, their impact on soybean oil/water interfacial tension was measured. The change in interfacial tension with time is shown in Figure 6, and the initial value of interfacial tension (γ_0_) of complex samples is shown in Table 2. Due to the adsorption of SPI and MCC/CMCC onto the interface, the interfacial tension decreased gradually with time. To better understand the differences, the interfacial tension was plotted against 1/$\sqrt{t}$, and the intercept of linear regression was taken as the steady-state O/W interfacial tension value at equilibrium (γ_∞_) [20]. As shown in Table 2, with the proportion of MCC or CMCC increased, γ_∞_ increased significantly. The data indicate that a high amount of MCC or CMCC was not helpful for the reduction of interfacial tension. In addition, complexes containing CMCC with higher cationization degree also showed larger γ_∞_ values than others, indicating the negative effect of positive charges on reducing surface tension. Therefore, the results show that interfacial tension-reducing ability was not the determining factor for the excellent emulsification ability of SPI-CMCC complexes, especially for SPI/CMCC-3 formula.

**Figure 7:**
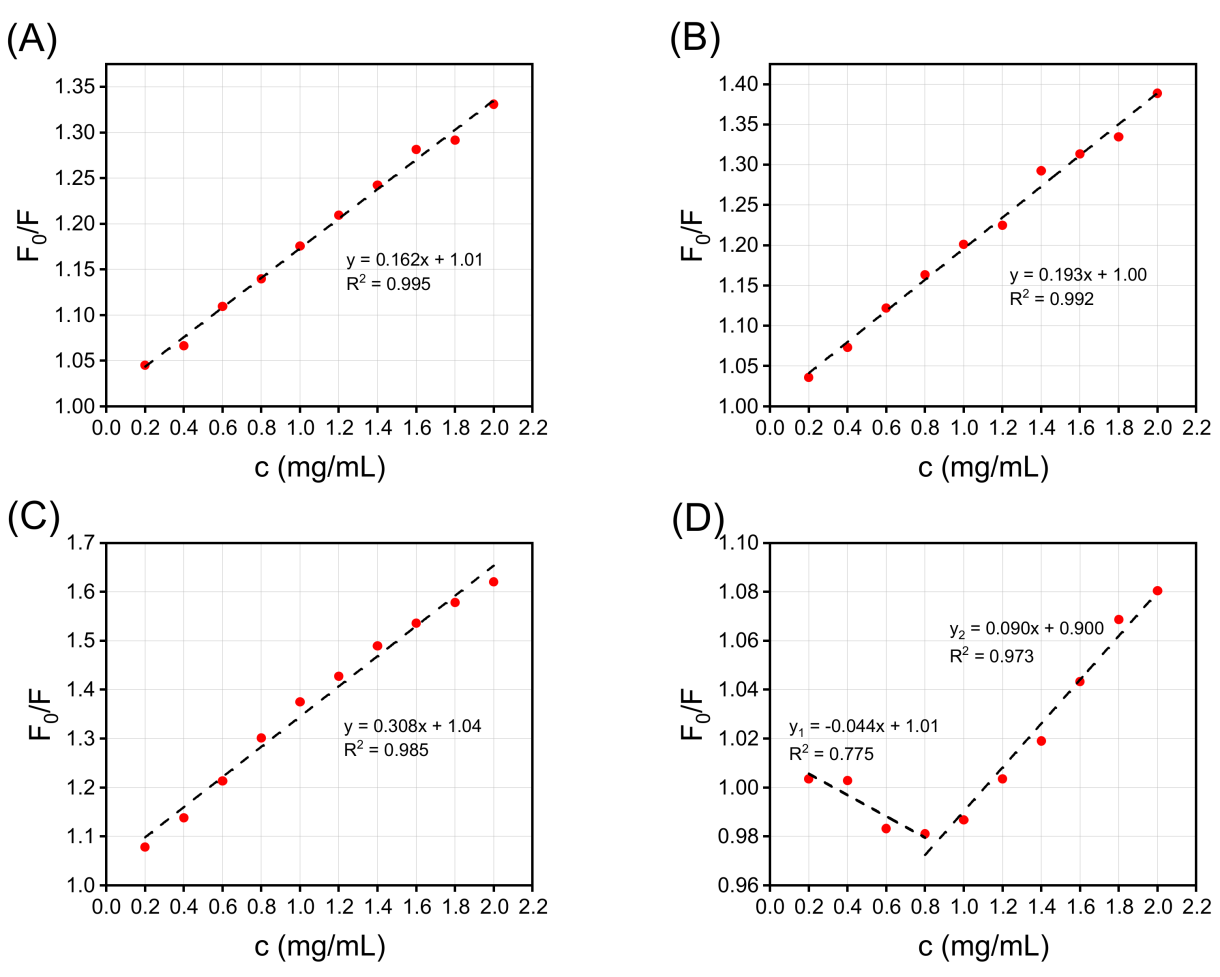
The effects of MCC (**A**), CMCC-1 (**B**), CMCC-2 (**C**), and CMCC-3 (**D**) on the fluorescence of 1 mg/mL SPI; the relationship between F_0_/F and c (MCC/CMCC) was analyzed on the basis of Equation (3), with the slope determined as the quenching constant *K*_SV_.

To better understand the interactions of SPI with MCC or CMCC, a fluorescence quenching method was applied, and the binding affinities between them were analyzed by using the Stern–Volmer equation (Figure 7). The fluorescence of SPI derived from tryptophan (*Trp*) residues was decreased gradually with the addition of MCC or CMCC, showing a quench effect, except for CMCC-3 [34,35]. The fluorescence intensity of SPI increased first with the addition of CMCC-3 and decreased rapidly when the concentration of CMCC-3 was greater than 0.8 mg/mL. This means that the interaction between CMCC-3 and SPI was different from other samples, and it was dose-dependent. For other samples, their *K*_SV_ values increased with the increase in cationization degree of CMCC, which means that the higher the cationization degree, the stronger the complexation ability of CMCC with SPI. However, the change in *K*_SV_ value of SPI with titration of CMCC-3 confirmed our previous conclusion that the interactions between them were different from the others. This critical point of fluorescence quenching existed at an SPI: CMCC-3 mass ratio of 1:1. As shown in Table 1, at this mass ratio, emulsions stabilized by SPI/CMCC-3 showed relatively low droplet size, with high zeta-potential value, indicating that the interaction between CMCC-3 and SPI dramatically changed at this critical point. However, more observations of the conformation changes are needed to better clarify the stabilization mechanism of this complex formula on emulsions.

**Figure 8:**
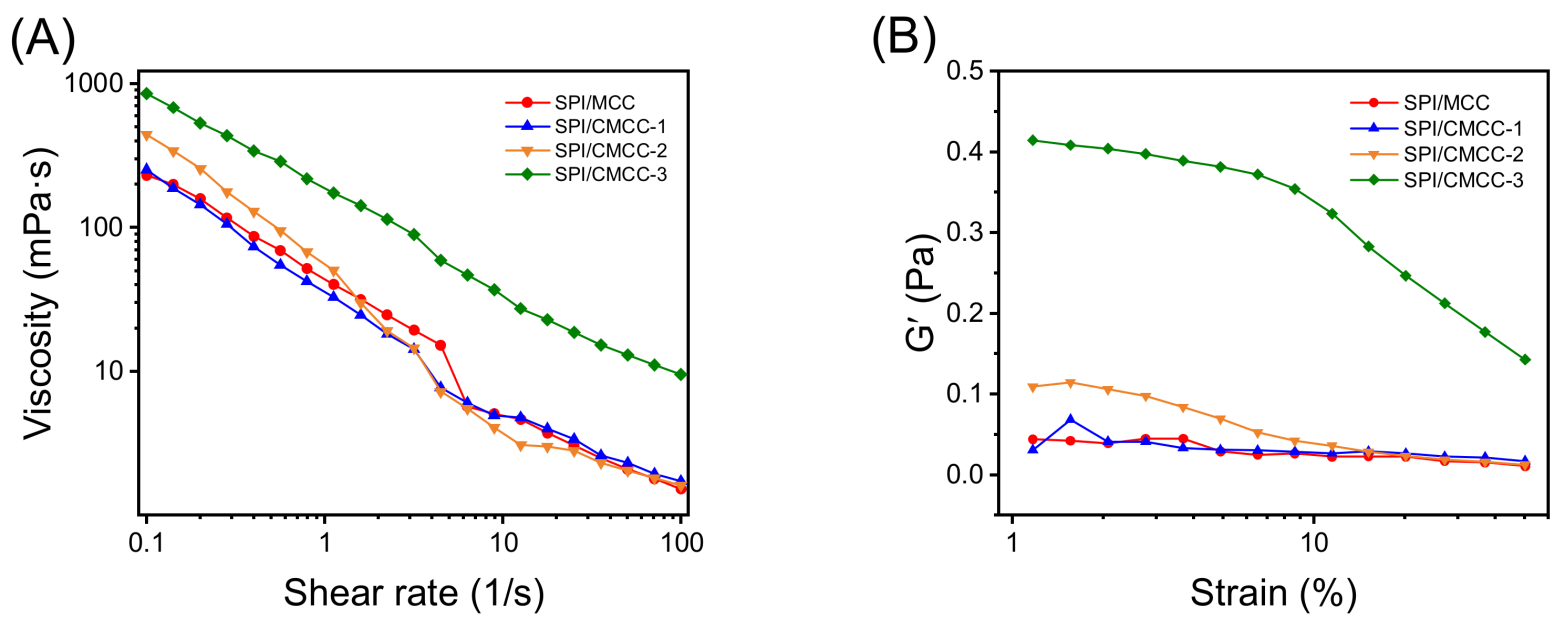
The viscosity (**A**) and the change in storage modulus (G') (**B**) of complexes for SPI and MCC/CMCC at ratio of 1:3 in a strain sweep test.

The rheological properties of SPI and MCC/CMCC complexes are shown in Figure 8. Although the viscosity of all complex samples decreased continuously with shear rate showing the shear thinning behavior, the magnitude of sample SPI/CMCC-3 was 10 times lower than that of CMCC-3 alone at the same mass concentration (Figure 3A), while the viscosity of complexes formed by SPI with MCC, CMCC-1, and CMCC-2 did not change significantly. The G’ values of all samples became smaller after compositing with SPI, indicating a reduced elasticity and mechanical strength, especially for sample SPI/CMCC-3. Even so, the viscosity and G’ of SPI/CMCC-3 complexes were significantly larger than those of other complex samples. Overall, the superior emulsifying property of SPI/CMCC-3 complexes may be attributed to the formation of a more viscoelastic network at O/W interface rather than simply providing electrostatic repulsion against droplet aggregations.

## 4. Conclusions

In this study, emulsions were prepared by complexes of SPI with MCC or cationic modified CMCC. The complexes formed between SPI and CMCC-3 showed the best emulsifying properties, and the emulsions stabilized by this composite formula at a ratio of SPI/CMCC at 3:1 were more stable than others. This complex formula showed completely different interaction mechanisms to other SPI/CMCC complexes, by providing a more viscoelastic network structure at the interface rather than simply reducing the surface tension. However, the interaction details should be further explored in future research. Our results may provide some theoretical basis for the preparation and application of SPI and polysaccharide complexes for food and cosmetic emulsions, especially for those require high stability.

## Figures and Tables

**Table 1:** The droplet size and zeta-potential of emulsions stabilized by SPI and MCC/CMCC complexes at mass ratios of 3:1, 1:1, and 1:3.

Ratio of SPI: MCC/CMCC		SPI/MCC	SPI/CMCC-1	SPI/CMCC-2	SPI/CMCC-3
3:1	*d*_43_ (µm)	60.70 ± 2.28 ^Bb^	50.81 ± 2.28 ^Bc^	45.76 ± 2.59 ^Bd^	97.06 ± 1.79 ^Aa^
	Zeta-potential (mV)	−41.1 ± 0.7 ^Ab^	−41.8 ± 0.9 ^Ab^	−40.9 ± 0.9 ^Ab^	−2.4 ± 0.3 ^Ca^
1:1	*d*_43_ (µm)	58.37.75 ^Ba^	50.05 ± 1.71 ^Bb^	46.99 ± 2.58 ^Bc^	24.81 ± 0.55 ^Bd^
	Zeta-potential (mV)	−44.0 ± 0.6 ^Bd^	−42.8 ± 1.1 ^Adc^	−41.4 ± 1.1 ^Ab^	45.5 ± 0.6 ^Ba^
1:3	*d*_43_ (µm)	69.86 ± 1.67 ^Aa^	68.40 ± 2.59 ^Aa^	55.39 ± 2.08 ^Ab^	20.25 ± 0.46 ^Cc^
	Zeta-potential (mV)	−45.3 ± 0.8 ^Cc^	−44.4 ± 0.9 ^Bc^	−27.9 ± 1.0 ^Bb^	51.5 ± 1.0 ^Aa^

Uppercase letters (A–C) mean significant differences of the same parameter within the same column, while lowercase letters (a–d) indicate horizontal comparisons of this parameter (*p* < 0.05).

**Table 2:** The initial interfacial tension (γ_0_) and steady-state interfacial tension at equilibrium (γ_∞_) of soybean oil/water interfaces as stabilized by complexes of SPI and MCC/CMCC at different ratios.

Ratio of SPI: MCC/CMCC	γ(mN/m)	SPI/MCC	SPI/CMCC-1	SPI/CMCC-2	SPI/CMCC-3
3:1	γ_0_	16.94 ± 0.30 ^Aa^	16.46 ± 0.07 ^Ba^	17.00 ± 0.31 ^Aa^	16.51 ± 0.18 ^Ba^
	γ_∞_	7.96 ± 0.17 ^Cc^	8.05 ± 0.07 ^Cb^	8.26 ± 0.14 ^Cb^	8.83 ± 0.01 ^Ba^
1:1	γ_0_	16.78 ± 0.30 ^Aa^	16.85 ± 0.17 ^ABa^	16.65 ± 0.27 ^Aa^	17.01 ± 0.08 ^ABa^
	γ_∞_	8.61 ± 0.09 ^Bab^	8.78 ± 0.04 ^Bab^	8.49 ± 0.12 ^Bb^	8.81 ± 0.27 ^Ca^
1:3	γ_0_	17.52 ± 0.04 ^Aa^	17.38 ± 0.41 ^Aa^	17.32 ± 0.32 ^Aa^	17.31 ± 0.34 ^Aa^
	γ_∞_	9.73 ± 0.05 ^Ab^	9.91 ± 0.14 ^Ab^	10.56 ± 0.03 ^Aa^	9.71 ± 0.17 ^Ab^

Means marked with different lowercase letters (a-c) indicate significant differences in the same row, while these with different uppercase letters (A-C) are significantly different within the same column (*p* < 0.05).

## Data Availability

The data presented in this study are available on request from the corresponding author.

## Supplementary Materials

The following supporting information can be downloaded at: https://www.mdpi.com/article/10.3390/foods11193100/s1; Figure S1: The loss modulus (G″) of MCC and CMCC in a strain sweep test.
