# Supplementary figures and images for: Effect of Cationic Modified Microcrystalline Cellulose on the Emulsifying Properties and Water/Oil Interface Behavior of Soybean Protein Isolate
Source: Foods. 2022 Oct 5;11(19):3100. doi: 10.3390/foods11193100 (PMC9562696; doi:10.3390/foods11193100)

# Supplementary data

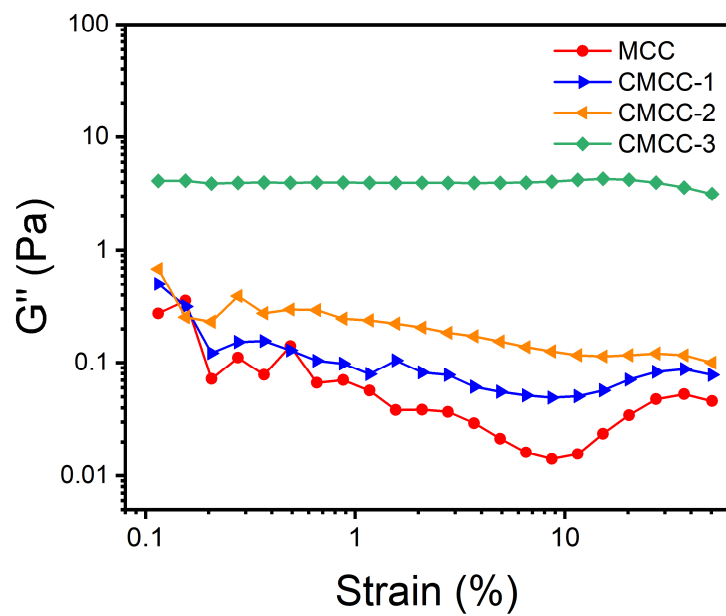

**Figure S1.** The loss modulus ( $G''$ ) of MCC and CMCC in a strain sweep test.

Supplement: Supplementary file 1 [file foods-11-03100-s001.zip › foods-1922902-supplementary.pdf]
